# Novel methods to monitor the biodegradation of polylactic acid (PLA) by *Amycolatopsis orientalis* and *Amycolatopsis thailandensis*

**DOI:** 10.3389/fbioe.2024.1355050

**Published:** 2024-04-09

**Authors:** Najwa Mat Yasin, Farlash Pancho, Md Yasin, Jan F. M. Van Impe, Simen Akkermans

**Affiliations:** ^1^ BioTeC+ - Chemical and Biochemical Process Technology and Control, KU Leuven, Ghent, Belgium; ^2^ Faculty of Ocean Engineering and Informatics, Universiti Malaysia Terengganu (UMT), Kuala Nerus, Terengganu, Malaysia

**Keywords:** *Amycolatopsis orientalis*, *Amycolatopsis thailandensis*, polylactic acid, aerobic PLA degradation, biomass, carbon balance

## Abstract

Plastics are essential in modern life, but their conventional production is problematic due to environmental pollution and waste management issues. Polylactic acid (PLA) is a widely used bioplastic that is bio-based and biodegradable, making it a key player in the bioeconomy. PLA has been proven to be degradable in various settings, including aqueous, soil, and compost environments. However, monitoring and optimizing PLA biodegradation remains challenging. This study proposes methods to improve the quantification of PLA biodegradation by *Amycolatopsis* spp. Ultrasound treatments (10 s) significantly improved the enumeration of viable *Amycolatopsis* cells by breaking the pellets into quantifiable individual cells. A separation technique combining ultrasound (120 s) and 40 μm cell strainers effectively isolated PLA particles from biomass to quantify PLA weight loss. This enabled the monitoring of PLA biofragmentation. Finally, CO_2_ production was measured according to ISO 14852 to quantify mineralization. Integrating these methods provides an improved quantification for PLA biodegradation along its different stages. In a case study, this led to the construction of a carbon balance where 85.1% of initial carbon content was successfully tracked. The developed techniques for monitoring of PLA biodegradation are essential to design future waste management strategies for biodegradable plastics.

## 1 Introduction

Plastics are essential in modern life, as they offer quality products for many uses. These sophisticated attributes of synthetic polymers have led to an annual plastic production of approximately 140 million tons (Teixeira et al., 2021). However, most conventional plastics are fossil-based, causing environmental pollution and waste management problems. The durability of plastics hampers their degradation in the biosphere, resulting in the long-term retention of plastic waste in the environment (Shimao, 2001; Mayekar et al., 2023). Chemical recycling is at its forefront as an economically viable technology for plastic waste management (JRC Technical Report, 2023). However, these processes are often energy intensive, for example, gasification requires a temperature up to 700°C (Shah et al., 2023). In response to these challenges, stakeholders have taken a paradigm shift to leverage bioplastics that are environmentally friendly. However, not all bioplastics are biodegradable, even if they are bio-based. Polylactic acid (PLA) is a widely used bioplastic in the market and accounts for 20.7% of global bioplastic production (European Bioplastic, 2022). Owing to its bio-based and biodegradable characteristics, it is one of the key players in the bioeconomy (Butbunchu and Pathom-Aree, 2019). PLA has been proven to be degradable in various controlled settings at the laboratory stage, including aqueous, soil, and compost environments (Apinya et al., 2015; Castro-Aguirre et al., 2017; Benn and Zitomer, 2018; Hobbs et al., 2019; Choe et al., 2022; García-Depraect et al., 2023; Mayekar et al., 2023). The *Amycolatopsis* genus, specifically *Amycolatopsis orientalis* and *Amycolatopsis thailandensis* are the most dominant PLA degraders in mesophilic conditions, which they accomplish by secreting extracellular enzymes such as lipases, esterases and cutinases to depolymerize ester bonds within the polymer (Li et al., 2008; Chomchoei et al., 2011; Butbunchu and Pathom-Aree, 2019).

However, most studies on bioplastics have utilized microbial consortia isolated from local waste compost, activated sludge from wastewater, dumping sites, and soil environments (Adhikari et al., 2016; Hottle et al., 2016; Hobbs et al., 2019; Mayekar et al., 2023). Due to their methodology based on the isolation of local bacteria, these studies are difficult to reproduce. Further optimization under controlled conditions, e.g., using pure microbial cultures and at specific temperatures, could pave the way for reproducible biological recycling. To optimize such a controlled biodegradation process, accurate monitoring of this process is essential. Therefore, to improve PLA biodegradation, it is required to be able to accurately monitor the growth of degrading microbes, the weight loss of the PLA and the production of biogas. Even though these measurements seem evident, their implementation for aerobic PLA degradation by *Amycolatopsis* species is still lacking from literature.

Both the quantification of viable cells and of PLA weight reduction are hampered by the specific growth morphology of bacteria from the *Amycolatopsis* genus. These bacteria form branched filamentous structures that resemble a fungal aerial mycelium. When grown on agar medium, these actinobacteria form branch fragmenting into squarish and rod-shaped elements (Tan and Goodfellow, 2015) and they exhibit pellet morphology in liquid medium. When using the traditional viable plate count method to quantify *Amycolatopsis* on agar media, the numerous aggregated cells of pellets are perceived as an individual colony forming unit. On the other hand, these pellets are difficult to separate from bioplastics as is required for the quantification of bioplastic weight loss.

This research proposes sonication treatment as the solution for both quantification problems. In microbiology, sonication is primarily used as an effective method for cell lysis and elimination of biofilms from surfaces (Ganesan et al., 2015; Dudek et al., 2020; Ferdous et al., 2021). The ultrasound mechanism works by the propagation of sound waves from the sonicator along the medium, which induces a distinctive pressure difference. This causes the formation of high-energy gas bubbles, also known as cavitations, with a localized temperature reaching 5000 K, which then quickly dissipates (Pitt and Ross, 2003). The collapse of these gas bubbles radiates a shockwave that can release the cells from the aggregated pellets into the planktonic form (Ganesan et al., 2015; Branck et al., 2017). The intensity of the ultrasound treatment is tuned by changing the amplitude, temperature, and treatment time. As such, sonication treatment was optimized in this study to balance the release of aggregated cells to make them quantifiable against the inactivation of cells (Dudek et al., 2020). With respect to the determination of PLA weight loss, the commonly used powder form in the range of 125–250 µm complicates the separation from the bacteria in the pellet morphology. This range of powder is commonly used as it is recommended by ISO 14 852 for plastic biodegradation studies. However, this PLA powder cannot be separated from bacterial pellets by centrifugation or filtration. Using the sonication method can form a solution by breaking the pellets into individual cells, making a separation based on particle size possible. Therefore, this research studies the combined use of sonication and filtration by cell strainers to determine the weight loss of PLA powder.

A complete biodegradation process of plastic involves either the determination of oxygen demand or carbon dioxide production (CO_2_) (and methane, CH_4_). The biodegradation step in which these processes occur, known as mineralization, determines the amount of polymeric carbon that is microbially converted into CO_2_ (or CH_4_) and biomass. Hence, monitoring of respirometry during biodegradation is crucial to monitor the biogas produced as end products (Choe et al. (2022). There is currently a wide array of techniques to measure CO_2_ evolution including direct measurement respirometry, gravimetric measurement respirometry and cumulative measurement respirometry (Ruggero et al., 2019). Owing to the slow CO_2_ evolution process for biodegradation of plastics, an established standard based on biogas cumulative measurement, ISO 14 852, was selected in this study. This method allows reliable, yet simple quantification of CO_2_ based on titrimetric measurements using barium hydroxide as a carbon capture solution.

In this study, we focus on developing a set of methodologies to monitor the degradation of PLA by *A. orientalis* and *A. thailandensis* in aqueous conditions using PLA powder ranging from 125 to 250 µm. Three methods have been proposed to improve the monitoring of the biodegradation process: 1) viable cell enumeration, 2) gravimetric weight loss of PLA plastics, and 3) cumulative biogas quantification. In the first methodology, low-frequency ultrasound (20 kHz, 50 W) was used to enumerate the viable cell growth of the microorganisms in their rich medium via process optimization using a series of short exposure times of 5, 10, and 15 s. To validate the sonication methods for cell enumeration, extracellular leakage was observed using the optical density at 260 nm. Parameter estimation analysis was performed to further characterize the differences in observing cell growth with and without sonication, also in the presence of PLA. Next, gravimetric PLA weight loss was conducted to measure the PLA weight loss caused by bacterial degradation. Because the biodegradation process involves a mixture of PLA powder and aggregated cells, mechanical separation using a cell strainer was adopted. To enable the separation of these particles, a sonication method was implemented to degrade aggregated cell pellets to smaller sizes that passed as filtrate through these cell strainers, separating them from the PLA in the retentate. Finally, the ISO 14 852 method was implemented to monitor CO_2_ production from PLA degradation to quantify the mineralization process. A simple carbon balance was calculated based on CO_2_ evolution. To the best of our knowledge, this is the first effort to enumerate the kinetic growth of *A. orientalis* and *A. thailandensis*, which will serve as stepping stones for controlled biodegradation of PLA in the future.

## 2 Materials and methods

**FIGURE 1:**
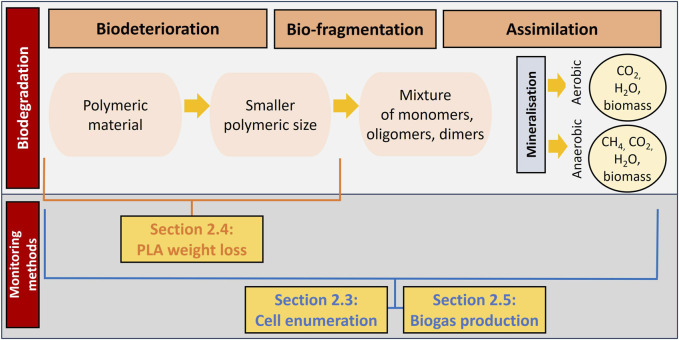
Representation of the link between the monitoring methods and the various phases of the biodegradation process (adapted from Yasin et al. (2022)).

The new methods that have been developed within this research are described in the sections below. Specifically, the developed method for the quantification of the viable cell population is explained in Section 2.3 and the new method for studying PLA weight loss is provided in Section 2.4. Moreover, the implementation of the ISO method for quantifying cumulative biogas production in the form of CO_2_ is provided in Section 2.5. The link between these three experimental methods and the biodegradation process is illustrated in Figure 1.

### 2.1 Strain and cultivation condition

*Amycolatopsis orientalis* and *Amycolatopsis thailandensis* were acquired from the NITE Biological Research Center (NBRC), Chiba, Japan. Storage and culturing of the strains were performed using yeast extract medium (YEM) containing 4 g yeast extract (Millipore, Merck, Darmstadt, Germany), 10 g malt extract (Carl Roth, Karlsruhe, Germany), 4 g glucose (Thermo Scientific, Kandel, Germany), and 20 g bacteriological agar (VWR International, Leuven, Belgium) per liter. Stock cultures of the strains were kept frozen at −80°C in YEM supplemented with 20% (v/v) glycerol (Acros Organics, Geel, Belgium), and thawed at room temperature prior to utilization. Purity plates for each microorganism were prepared by spreading the stock on YEM agar plates, containing 20 g/L agar (NBRC), and incubating at 30°C for 5 days. Precultures were prepared by transferring one colony from the purity plate into 75 mL of freshly prepared YEM broth, which was incubated at 30°C for 5 days. The preculture was cultivated in 100 mL baffled shake flasks while shaken at 180 rpm until the cells reached the stationary phase.

In the biodegradation experiments, where PLA was used as the sole carbon source, following cultivation in rich medium, the cells were pelleted by centrifugation (4629 g for 20 min) and resuspended in minimal medium (MM). Minimal medium composition per liter was 200 mg magnesium sulphate heptahydrate (MgSO_4_.7H_2_O), 1600 mg potassium phosphate dibasic (K_2_HPO_4_), 200 mg monopotassium phosphate (KH_2_PO_4_), 1000 mg ammonium sulphate ((NH_4_)_2_SO_4_), 100 mg sodium chloride (NaCl), 20 mg calcium chloride dihydrate (CaCl_2_.2H_2_O), 0.5 mg sodium molybdate dihydrate (Na_2_MoO_4_.2H_2_O), 0.5 mg sodium tungstate dihydrate (Na_2_WO_4_.2H_2_O), 0.5 mg manganese sulphate (MnSO_4_) and 25 mg ferrous chloride (FeCl_2_.4H_2_O). All chemicals were prepared as stock solutions under sterile conditions and were of analytical grade. MM was freshly prepared prior to each experiment by adding a proportionate amount of stock solution into sterile distilled water under continuous stirring. The pH of the minimal medium was 7.5.

### 2.2 Bioplastics

The PLA plastics used in this paper were IngeoTM 2003D (NatureWorks ^©^ LLC, Minnetonka, MN, United States) and Futerro PLA was provided by Futerro S.A. (Escanaffles, Belgium). These bioplastics were originally in granulated form. Prior to all experimental work, the PLA bioplastics were processed to transform the granules into powder (Choe et al., 2022; García-Depraect et al., 2022). PLA granules (100 g) were ground together with alternate layers of dry ice (200 g, IJsfabriek, Belgium) using a commercial blender (Krups Blender, Prep Expert S7000; 1,000-W). A total of 15 grinding cycles (5 min grind; 3 min rest) was used to avoid overheating of the appliance and reduce sublimation.

The grounded PLA powder was dried overnight at room temperature. Finally, manual sieving using stainless steel sieves of 100, 125, 150, and 250 µm (Test Sieve ISO 3310/1, Fisherbrand) was conducted to classify the powders based on particle size. The particle size used was from 125 to 250 µm as recommended by ISO 14 852 for aerobic aqueous conditions. These particles were sterilized with 70% ethyl alcohol prior to working under aseptic conditions.

### 2.3 Viable cell enumeration

#### 2.3.1 Sonicator treatment for quantifying viable cells

Sonication was implemented to decompose the flocs formed by *A. orientalis* and *A. thailandensis* into planktonic cells for enumeration. Experiments were conducted in 75 mL YEM in baffled shake flasks at 180 rpm and 30°C for 5 days. Samples of 900 µL were taken regularly and sonicated at 100% amplitude for 5, 10 and 15 s in a 1 mL Eppendorf tube using a Fisherbrand Model 50 Sonic Dismembrator with 1/8” probe. Samples were then serially diluted and plated on YEM agar (100 µL sample per plate for each dilution). Each sample was taken in quadruplicate and a comparison was made with samples without sonication. Agar plates were incubated at 30°C for 5 days. Similar experiments were conducted to monitor growth of both strains in a setting for biodegradation, where PLA served as the sole carbon source. Considering longer degradation for PLA would take place, this experiment lasted for 14 days.

#### 2.3.2 Growth characteristics

The growth characteristics were quantified by performing a parameter estimation with the model of Baranyi and Roberts (1994) on the experimental data. This parameter estimation led to the identification of the initial population density ( $N_{0}$ ), the lag phase duration ( λ ), the maximum specific growth rate ( $\mu_{\max}$ ) and the maximum population density ( $N_{\max}$ ). The model equations are formulated as follows:

$$\frac{\text{dN}\left(t\right)}{\text{dt}}=\frac{Q\left(t\right)}{1+Q\left(t\right)}\cdot \mu_{\max}\cdot \left(1-\frac{N\left(t\right)}{N_{\max}}\;\right)\cdot N\left(t\right)\text{ with }N\left(t=0\right)=N_{0}$$

$$\frac{\text{dQ}\left(t\right)}{\text{dt}}=\mu_{\max}\cdot Q\left(t\right)\text{ with }Q\left(t=0\right)=Q_{0}$$

$$\lambda =\frac{\ln\left(1+1/Q_{0}\right)}{\mu_{\max}}$$

where Q is the physiological state of the cell and its initial condition is $Q_{0}$ . This physiological state is used to describe the lag phase of the bacteria. The model parameters were estimated using the function *lsqnonlin* of MATLAB (MathWorks) while the viable cell counts were transformed as follows: $n\left(t\right)=\ln\;\left(N\left(t\right)\right)$ , to stabilize the variance (Smet et al., 2017).

#### 2.3.3 Effect of sonication on viability

To examine the applicability of sonicator treatments for cell enumeration, the integrity of sonicated *A. orientalis* and *A. thailandensis* cells was evaluated by measuring the leakage of intracellular constituents to the supernatant (Huang et al., 2019; Umair et al., 2022). Precultures (40 mL) were prepared in YEM, inoculated and stored shaken at 180 rpm for 5 days at 30°C. On the last day, the preculture was resuspended in 8 g/L sodium chloride solution after centrifugation at 13,600 g for 15 min. For each strain and treatment time, 1 mL of preculture was transferred into a sterile 1.5 mL Eppendorf tube three times. The tubes were subjected to sonication for 5, 10, 15, 60 and 120 s. The samples were centrifuged at 10,000 g for 10 min (4°C), and the supernatant of three identical tubes was transferred into a 3 mL quartz cuvette to examine the release of extracellular constituents at 260 nm (VWR UV-6300PC, Haasrode, Belgium). Samples were prepared in triplicate, with untreated samples as controls.

### 2.4 Gravimetric weight loss of PLA bioplastics

#### 2.4.1 Optimization of PLA and cell separation following inoculation

Determining the weight loss of PLA powder is complicated by the bacterial flocs that are difficult to separate from this powder. In this study, a new method was developed to separate PLA powder from the bacteria by sonicating samples followed by filtration with 40 μm cell strainers (nylon mesh, Fisherbrand). Optimization studies were conducted to compare the percentage of PLA recovery at sonication times of 30, 60, 120, 180, and 240 s. All cell strainers were pre-weighed and covered with aluminum foil to keep them clean. First, 4 g/L of PLA powder was re-suspended in a 10 mL preculture combined with 20 mL MM in a 50 mL Falcon tube (Cellstart^®^, Greiner Bio-Lab, Vilvoorde, Belgium). The Falcon tube was placed in an ice bath during sonication. Sonicated samples were filtered by pouring them through the cell strainer and the cell strainer was rinsed 5 times with 1 mL of ultrapure water using a micropipette. Used cell strainers were dried at 55°C for 24 h and weighed again to determine the amount of PLA powder in the retentate. All samples were prepared in five replicates.

#### 2.4.2 PLA biodegradation using *Amycolatopsis orientalis* and *Amycolatopsis thailandensis*

To validate the developed method for quantifying PLA weight loss, a PLA degradation study was implemented to compare biodegradation of two PLA types (Futerro and NatureWorks) by both microorganisms. The preculture was directly inoculated in the system. Centrifuge tubes containing 40 mL of preculture were sonicated at an amplitude of 100 for 10 s to homogenize them. Then, 1 mL was transferred to a disposable cuvette (Polystyrene, Fisherbrand™) to determine the absorption at 595 nm. The measured optical density ${\text{OD}}_{i}$ was used to determine the inoculation volume $V_{i}$ required to achieve the same cell density after inoculation according to the following equation:

$$V_{i}=\frac{V_{r}\cdot {\text{OD}}_{r}}{{\text{OD}}_{i}}$$

In this equation the reference volume $V_{r}$ and reference optical density ${\text{OD}}_{r}$ were fixed to 10 mL and 0.173. Petri dishes ( ∅ 90 Gosselin, Fisher Scientific) were filled with 120 mg of PLA, $V_{i}$ mL of inoculum and 30- $V_{i}$ mL of minimal medium and incubated at 30°C.

On the fourth week of inoculation, Petri dishes were removed from the incubator. The content from each Petri dish was transferred to a 50 mL centrifuge tube and any residue was removed from the Petri dish by adding 5 mL ultrapure water and using a cell scraper, after which it was added to the centrifuge tube. The PLA degradation was then quantified using the method in Section 2.4.1 using a treatment time of 120 s. Gravimetric weight loss for the residual PLA was determined by weighing the PLA samples (KERN Precision balance, EWJ 300–3). In this way, the residual PLA in the samples, which determines the extent of degradation, could be measured. The weight loss of PLA was determined by using the equation below. Blank samples were prepared for both types of bioplastics using only minimal medium and PLA powder without bacteria. All samples were prepared in four replicates.

$$\text{PLA loss }\left(\%\right)=\frac{\left(W_{i}-W_{f}\right)}{W_{i}}\times 100$$

where Wi is the initial weight of the PLA powder g) and W_f_ is the final weight (i.e., after degradation) g).

### 2.5 Cumulative biogas quantification via CO_2_ evolution

**FIGURE 2:**
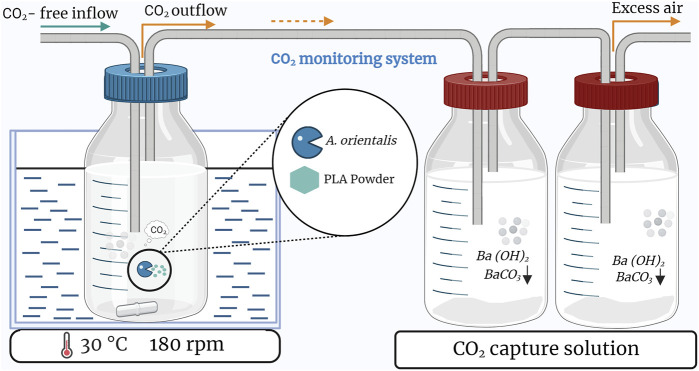
Schematic of the setup used to monitor biodegradation based on carbon dioxide production in a single bioreactor. Triplicate samples are based on three of these setups in parallel.

The PLA biodegradability was assessed following the ISO 14852 standard. The biogas production from the aerobic biodegradation was monitored by determining cumulative CO_2_ production. Therefore, an experimental system was used consisting of: 1) a controlled flow of CO_2_-free air, 2) a bioreactor and 3) a CO_2_ scrubbing system (Baldera-Moreno et al., 2022; Choe et al., 2022), as illustrated in Figure 2. This biodegradation setup was implemented to monitor the CO_2_ evolution by *A. orientalis* using two different carbon substrates: glucose (4 g/L) and PLA powder (1 g/L, 120–250 µm), for 7 and 30 days, respectively. All samples consisted of 200 mL MM and were inoculated with similar inoculum concentrations, as described in Section 2.4.2 with V_r_ and OD_r_ fixed to 10 mL and 0.173 (in triplicate). The duplicate blanks contained only the MM and cells. CO_2_-free air with less than 0.1 ppm-mol (ALPHAGAZ™ 2, Air Liquide) was supplied through a 0.2 µm filter at a flowrate of 0.05 lpm for each bioreactor using a flow meter. The biodegradability test was performed in 250 mL bottles equipped with a multi-inlet cap, stirred at 180 rpm and heated to 30 °C in a water bath (IKA IBR RO 15, Fisher Scientific). The CO_2_ biogas was transferred directly into a gas washing bottle filled with 300 mL of barium hydroxide solution (12.5 mM) according to this reaction. This led to the following reaction that decreases the pH of the barium hydroxide solution:

$${\text{Ba}\left(\text{OH}\right)}_{2}+{\text{CO}}_{2\;}\to \;{\text{BaCO}}_{3}↓+H_{2}O$$

Two CO_2_ scrubbing bottles were placed in series on the outflow of every bioreactor bottle. At the time of sampling, the first bottle was replaced by the second one and a fresh bottle was added. A 40 mL sample was taken from the first barium hydroxide bottle and filtered through 0.45 µm membrane filter (Filtropur S PES, Sarstedt) to remove precipitate. The solution was titrated to pH 7 with 50 mM HCl using a pH meter (Mettler Toledo, ECOLAB):

$${\text{Ba}\left(\text{OH}\right)}_{2}+2\text{ HCl}\to \;{\text{BaCl}}_{2}+2\;H_{2}O$$

The amount of HCl needed to achieve the equivalence point was measured to determine the quantity of CO_2_ that was captured:

$$n_{CO_{2}}=n_{\text{Ba}{\left(\text{OH}\right)}_{2}}-\frac{n_{\text{HCl}}}{2}$$

where $n_{CO_{2}}$ is the quantity of CO_2_ captured, $n_{\text{Ba}{\left(\text{OH}\right)}_{2}}$ is the original quantity of Ba(OH)_2_ in the scrubbing bottle and $n_{\text{HCl}}$ is the quantity of HCl that would be required to neutralize this entire bottle to pH 7. The mineralization (%) is calculated based on the amount (in mg or mole) of CO_2_ captured in the sample ( ${\text{CO}}_{2,S}$ ) and in the blank ( ${\text{CO}}_{2,B\;}$ ) compared to the theoretical amount of ${\text{CO}}_{2}$ produced when fully oxidizing the carbon substrate ( ${\text{CO}}_{2,T}$ ):

$$\text{Mineralization }\left(\%\right)=\frac{{\text{CO}}_{2,S}-\;{\text{CO}}_{2,B\;}}{{\text{CO}}_{2,T}}\cdot 100$$

At the end of the experiment, the PLA weight loss was determined according to the method of Section 2.4 with a sonication time of 120 s by distributing the bioreactor content over 3 centrifuge tubes of 50 mL. Biomass was measured based on dry cell weight determination after centrifugation. A carbon balance was determined to track the fate of carbon content conversion along the biodegradation process chain using carbon fraction that 1) converted to CO_2_, 2) converted to biomass (calculated based on the average biomass molecular formula of CH1.7O0.4N0.2 according to Popovic (2019)), 3) undegraded PLA and 4) unaccounted residual PLA.

## 3 Results and discussion

In this paper, three methodologies were developed to enhance the monitoring of polylactic acid (PLA) degradation in aqueous media by utilizing *A. orientalis* and *A. thailandensis*. These methodologies are linked to the various stages of biodegradation as illustrated in Figure 1 and include monitoring 1) viable cell enumeration, 2) gravimetric weight loss of PLA plastics, and 3) cumulative biogas quantification. Following these monitoring methods, the carbon balance was computed.

### 3.1 Viable cell enumeration

To evaluate the performance of *A. orientalis* and *A. thailandensis* in the degradation of PLA under controlled conditions, it is crucial to employ appropriate enumeration techniques to monitor cell growth. To this end, a sonication method is developed in this section. The developed technique was validated by measuring the cell membrane integrity at 260 nm. This sonication method was limited to improving the cell quantification and was applied directly after taking sample. As such, it was not used in biodegradation process itself and therefore did not affect the degradation process or the final carbon balance.

#### 3.1.1 Optimizing sonication for quantifying viable cells growth

**FIGURE 3:**
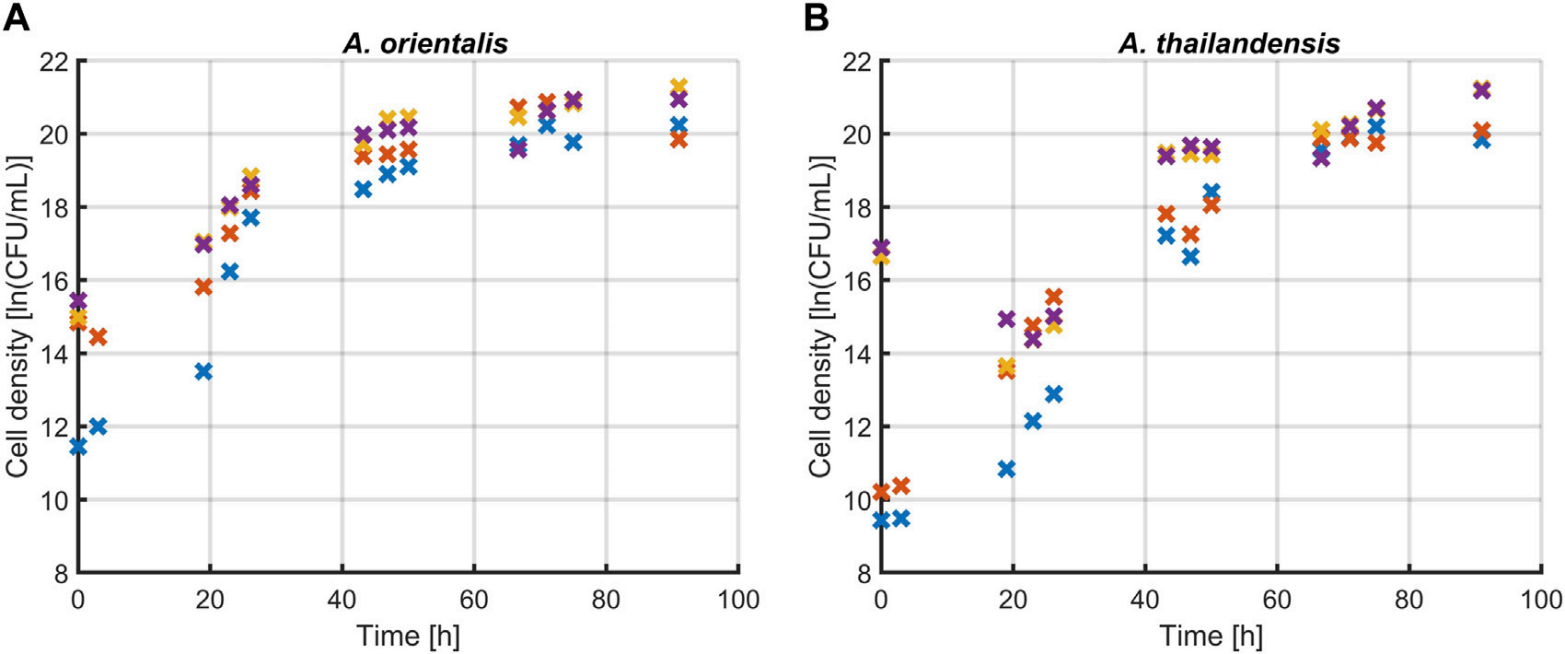
Effect of sonication on the quantification of viable **(A)** *Amycolatopsis orientalis* and **(B)** *Amycolatopsis thailandensis* cells through plate counts. The effects of 5 (**x**), 10 (**x**) and 15 (**x**) s of sonication were compared to a control without sonication (**x**). Data points are the averages of four technical replicates.

The first step was to determine the effect of sonication treatments on the quantification of *Amycolatopsis* spp. during population growth. Figure 3 presents the comparison between three different treatment times (5, 10 and 15 s) and untreated controls for both *A. orientalis* and *A. thailandensis*. The results were analyzed with a two-way ANOVA (*p* < 0.05) that considers treatment time and culturing time. For *A. orientalis*, this analysis demonstrated that treating samples at any sonication time led to significantly higher counts compared to untreated samples. On the other hand, there was no statistical difference between the three different treatment times for this strain. In the case of *A. thailandensis*, there was no significant effect of treating the samples for 5 s compared to untreated samples. Only when treating samples for 10 or 15 s, a significant increase in the cell count was found. Also, for *A. thailandensis*, there was no significant difference between these two treatments. When combining these sonication results, it is concluded that a minimum sonication time of 10 s is required to obtain a statistically significant improvement in the quantification of *A. spp*. Figure 3 also indicates that the increase in cell recovery due to sonication differs during different phases of cell growth. The effect of sonication treatment on the characterization of the various phases of growth is discussed in Section 3.1.2. On average, the quantity of viable cells that was detected (before logarithmic transformation) increased 7-fold for *A. orientalis* and 6-fold for *A. thailandensis* compared to untreated samples.

The sonication treatment successfully improved the quantification of the total viable population by releasing individual cells from agglomerates. When culturing *A.* spp., cells agglomerate in the form of dense pinpoint flocs (Tan and Goodfellow, 2015). These flocs appear as single colony forming units when applying plate count methods. The ultrasound waves of the sonication treatment apply strong shear forces on these flocs that led to the release of agglomerated cells into the planktonic form. This was visibly verified during the experiments as flocs were no longer observed after the applied sonication treatments. Ultrasound has been shown to effectively detach cells, which in this case resulted in an increase in the number of enumerated cells (Huang et al., 2017). Previous research has demonstrated that the use of sonication has a combined deagglomerating and inactivating effect. The research of Joyce et al. (2011) found these effects to be frequency dependent. In their research, low frequencies such as 20 and 40 kHz were causing a decrease in cell viability, rather than an increase in cultivability as seen in this study. This difference could be due to the fact that Gram-negative bacteria were studied in Joyce et al. (2011) whereas *A. orientalis* and *thailandensis* are Gram-positive.

#### 3.1.2 Effect of sonication on evaluating growth kinetics

**FIGURE 4:**
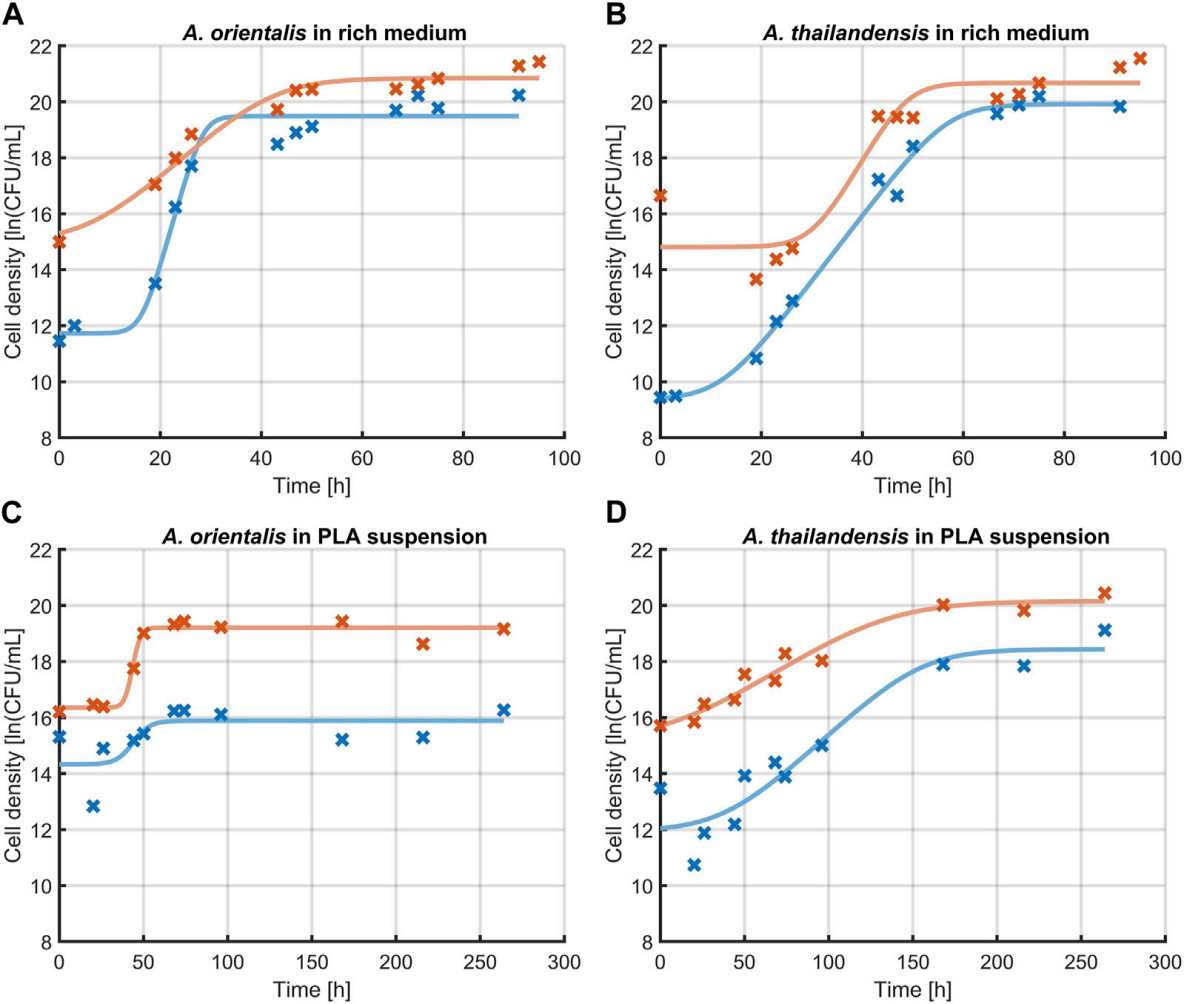
Effect of sonication on the measurement of growth kinetics of *Amycolatopsis orientalis* **(A,C)** and *Amycolatopsis thailandensis* **(B,D)** in rich medium **(A,B)** and PLA suspension **(C,D)**. A comparison is made between cell quantification without sonication (**x, —**) and after 10 s of sonication (**x, —**). Data points (x) have been fitted with the growth model of Baranyi and Roberts (1994) (−).

The effect of sonication on the characterization of growth kinetics was studied. Figure 4 illustrates the change in microbial growth kinetics as determined with or without sonicating the samples before quantification through viable plate counts. Specifically, the effect of 10 s of sonication was evaluated on the determination of the initial population density, the lag phase duration, the maximum specific growth rate in the exponential phase and the maximum population density that is reached in the stationary phase. These characteristics of microbial growth were determined by fitting the model of Baranyi and Roberts (1994) on the experimental data that was obtained and significant differences were analyzed between the model parameters that quantify each of these growth characteristics. The model parameters are compared in Table 1. The comparison was made for *A.* spp. growing in a rich medium and in PLA that was suspended in minimal medium (without any other carbon source aside from PLA). In all cases, the growth curve that was found through sonicating samples had higher population densities compared to growth curves from the untreated control. This increase in the measured cell densities is demonstrated by the initial cell densities and maximum cell densities that are consistently significantly higher when using ultrasound treated samples.

**TABLE 1:** Evaluation of the growth characteristics of *Amycolatopsis orientalis* and *Amycolatopsis thailandensis* as evaluated without sample treatment (control) compared to using 10 s of sonication before diluting and plating. Different superscript indices between parameters of the control and sonicated samples indicate statistical differences (*p* < 0.05).

	*A. orientalis*			
	Rich medium		PLA	
	Control	10 s sonication	Control	10 s sonication
$n_{0}$ [ln (CFU/mL)]	11.7 ± 0.4^a^	15.3 ± 0.4^b^	14.3 ± 0.5^a^	16.3 ± 0.2^b^
λ [h]	16.3 ± 1.7^a^	8.0 ± 5.6^b^	40.2 ± 21.2^a^	38.8 ± 3.9^a^
$\mu_{\max}$ [1/h]	0.64 ± 0.15^a^	0.16 ± 0.04^b^	0.21 ± 0.61^a^	0.30 ± 0.16^a^
$n_{\max}$ [ln (CFU/mL)]	19.5 ± 0.2^a^	20.8 ± 0.2^b^	15.9 ± 0.3^a^	19.2 ± 0.1^b^

	*A. thailandensis*			
	Rich medium		PLA	
	Control	10 s sonication	Control	10 s sonication
$n_{0}$ [ln (CFU/mL)]	9.4 ± 0.3^a^	14.8 ± 0.7^b^	12.0 ± 0.7^a^	15.7 ± 0.4^b^
λ [h]	12.3 ± 2.3^a^	30.3 ± 12.2^b^	40.6 ± 31.9^a^	20.8 ± 25.6^a^
$\mu_{\max}$ [1/h]	0.24 ± 0.02^a^	0.32 ± 0.24^a^	0.06 ± 0.03^a^	0.04 ± 0.01^a^
$n_{\max}$ [ln (CFU/mL)]	19.9 ± 0.2^a^	20.7 ± 0.4^b^	18.4 ± 0.7^a^	20.2 ± 0.3^b^

Symbols: $n_{0}$ , initial population density; λ , lag phase duration; $\mu_{\max}$ , maximum specific growth rate in the exponential phase; $n_{\max},$ maximum population density that is reached in the stationary phase.

In the case of cell growth in PLA suspensions, there were no significant differences in the lag phase duration or the exponential growth rate. As such, the use of the sonication treatments leads to an upwards shift of the growth curve. This represents the increased detection of individual cells by applying sonication before further sample processing during viable plate counts.

When looking at the growth parameters of the *A.* spp. in rich medium, also the lag phase duration and exponential growth rate are defined differently when using sonication. These differences are caused by the fact that the increase in cell recovery is not equal in all phases of growth. The same phenomenon is seen to a lesser extent for cells growing in a PLA suspension. Given that the sonicated samples lead to an improved quantification of the individual cells, the respective characterization of the growth phases is considered more representative compared to untreated samples.

#### 3.1.3 Effect of sonication on cell membrane integrity

**FIGURE 5:**
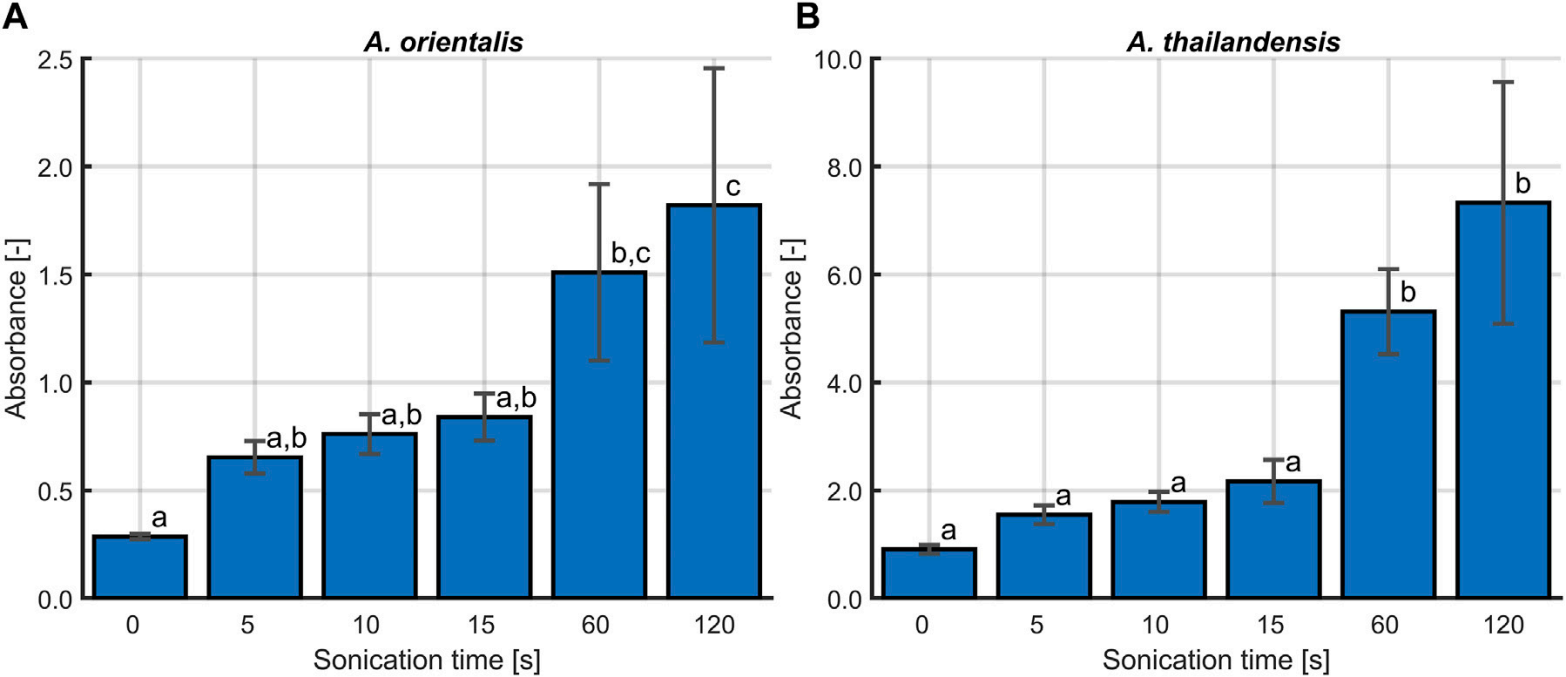
The effect of sonication time on the cell membrane integrity for **(A)** *Amycolatopsis orientalis* and **(B)** *Amycolatopsis thailandensis*. The membrane integrity is quantified by determining the amount of cell leakage, which is proportional to the absorbance of the supernatant at 260 nm. Bars bearing different letter indices have a statistically different mean (*p* < 0.05).

The last part of the evaluation of the sonication method was to determine the effect of these treatments on the cell membrane integrity. The cavitations that lead to the disaggregation of flocculated *A.* spp. are known to cause damage to the bacterial cell membrane. Li et al. (2016) found that ultrasound causes damage on the cytoplasmic membrane of Gram-positive bacteria. This membrane damage leads to leakage of essential intracellular components such as DNA. Depending on the amount of damage, cells will transition into a non-viable or non-culturable state, causing them to be undetectable with the viable plate count method (Böllmann et al., 2016). Therefore, the membrane damage caused by sonication was studied for both *A. orientalis* and *A. thailandensis*. Membrane damage was quantified by measuring the amount of leakage of cytoplasmic components based on the absorbance of the supernatant at 260 nm (Chen and Cooper, 2002). The results of these absorbance measurements after treatments of 0, 5, 10, 15, 60 and 120 s are illustrated in Figure 5. The longest treatment times of 60 and 120 s were included as cases with a high degree of membrane damage for comparison. In the case of treatments of 120 s, no viable cells could be detected. As such, this can be considered as a reference for membrane damage when all cells are inactivated to a non-viable or non-culturable state. For both strains, it is seen that there is a significant increase in the cell leakage when long treatments of 60 or 120 s are applied. In the case of short treatments of 5–15 s, there appears to be an increase in cell leakage for both strains, but this was not found to be statistically significant. As such, it is concluded that the use of sonication at the current conditions leads to membrane damage, which causes part of the population to become non-viable or non-culturable. However, in case of short treatments, there is a much higher increase in the quantity of colony forming units due to disaggregation of flocs than there is a decrease in viability or culturability. As such, short sonication times are beneficial to improve the accuracy of viable plate counts and the selected treatment time of 10 s leads to a desirable trade off between a significant amount of disaggregation and an insignificant amount of membrane damage.

### 3.2 Gravimetric weight loss of PLA

The quantification of degraded PLA samples is an important parameter in biodegradation. Separation between the PLA samples and cells is crucial prior to measuring the polymer weight loss of degraded samples. In this study, a PLA powder of 125–250 µm was used in an aqueous medium. Based on these powders, a separation method was first developed and then validated based on a case study in which the biodegradation of PLA by *A. orientalis* and *A. thailandensis* was compared for PLA from two different manufacturers.

#### 3.2.1 Optimization of PLA and cells separation

**FIGURE 6:**
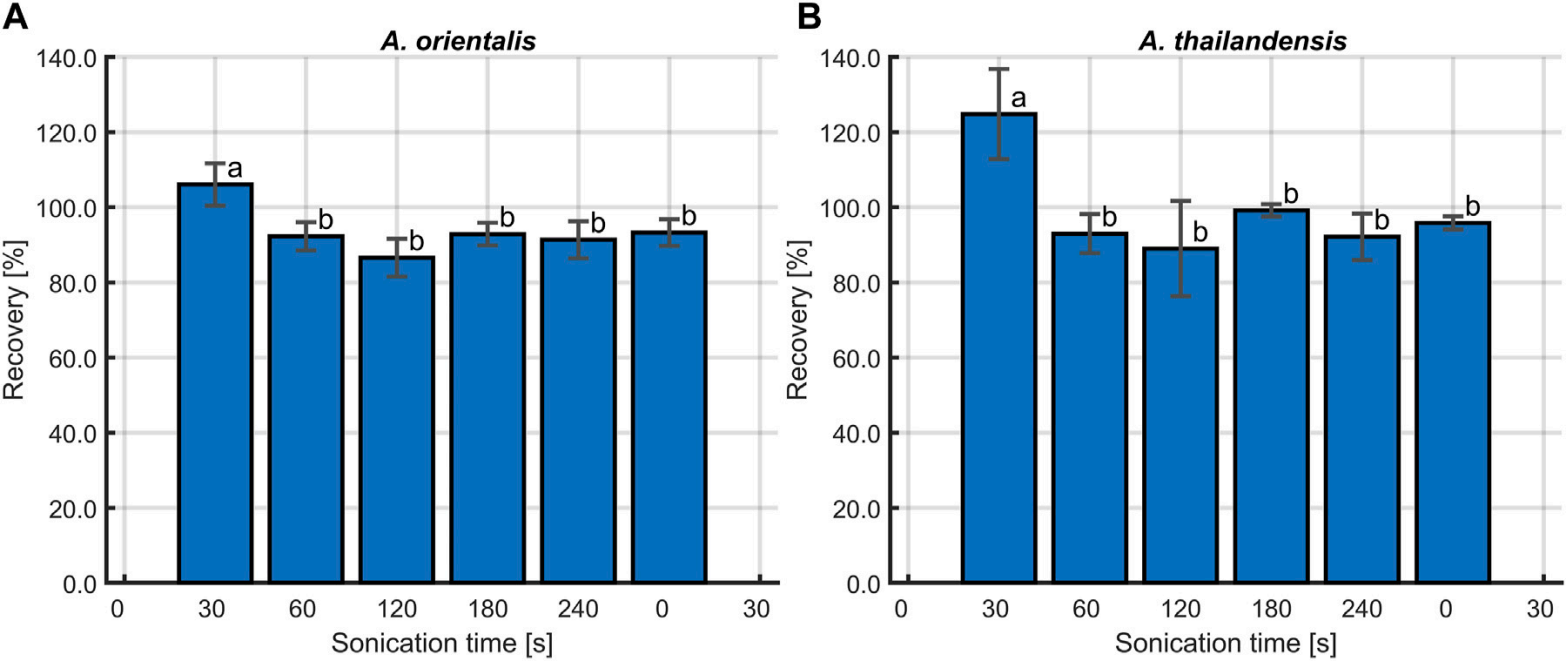
Percentage of PLA recovery at different sonication times, following inoculation with **(A)** *Amycolatopsis orientalis* and **(B)** *Amycolatopsis thailandensis.* Bars bearing different letter indices have a statistically different mean (*p* < 0.05).

A separation method was implemented that relied on sonication to disrupt cell agglomerates and to remove cells that are attached to the polymer surface. The individual planktonic cells are then separated from the larger PLA particles by using a cell strainer of 40 µm. This method was optimized on samples containing *A. orientalis* and *A. thailandensis* with the PLA powder. The percentage of PLA recovery in untreated samples was compared with samples treated by sonication for 30, 60, 120, 180, and 240 s. As shown in Figure 6, untreated samples had a recovery rate of about 125%. This overestimation of the recovery rate above 100% indicates the inclusion of cell pellets in the measured weight. The retention of agglomerated cells was visually confirmed when analyzing the dried strainers. Statistical analysis using one-way ANOVA (*p* < 0.05) revealed that all sonication treatments had a significant effect on recovery compared to the untreated samples. As such, the ultrasound waves were efficient in deagglomerating cells to a size that was sufficiently small to pass through the cell strainer. On the other hand, there was no significant difference between the percentage of PLA recovery for any of the sonication treatment times for both *A. orientalis* and *A. thailandensis.* As such, it appears to be unlikely that an increased sonication time would lead to mechanical degradation of the PLA powder and would cause an overestimation of the biodegradation. As such, a sonication time of 120 s was selected to decrease the risk of overestimations of the PLA weight under conditions that would lead to stronger attachment or aggregation of cells.

The weight loss measurement requires an effective separation technique to retrieve polymer residue from samples containing biomass prior to weighing (Shah et al., 2008). The traditional separation methods such as centrifugation and filtration that were commonly employed to measure degraded plastics in the form of films or sheets in aqueous environments (Ayar-Kayali and Tarhan, 2007; Grause et al., 2022; Samat et al., 2023) were not effective when suspension of cells and PLA powders were used. The combination of a cell strainer and sonication proposed in this method has proven to be effective to separate cells and PLA powders at any treatment time tested compared to untreated sample.

When the PLA powder is reduced in size to particles smaller than the cell strainer pore size, i.e., 40 μm, the PLA will pass through, indicating that the first stage of degradation (biodeterioration) has occurred. When assuming a spherical size of the PLA powder, particles of 125–250 µm in diameter would have to be degraded respectively over 96% and 99% in volume to be able to pass through the 40 μm cell strainers. As such, this method enables the quantification of the biofragmentation step by monitoring PLA weight loss. This new method for the quantification of plastic biodegradation is a new application for cell strainers, which have already found applications in various fields (Qiu et al., 2018; Takino et al., 2018; Wang et al., 2019). To the authors’ knowledge, this is the first study reporting on the combined use of cell strainers and sonication to analyze the biodegradation of plastics. A previous study has determined the residual quantity of PLA using a dialysis bag with at molecular weight cut off of 10 kDa (Youngpreda et al., 2017). However, given the small pore size, such a dialysis bag would not be suitable in the current application to separate biomass from PLA.

#### 3.2.2 PLA biodegradation by *Amycolatopsis orientalis* and *Amycolatopsis thailandensis*

**FIGURE 7:**
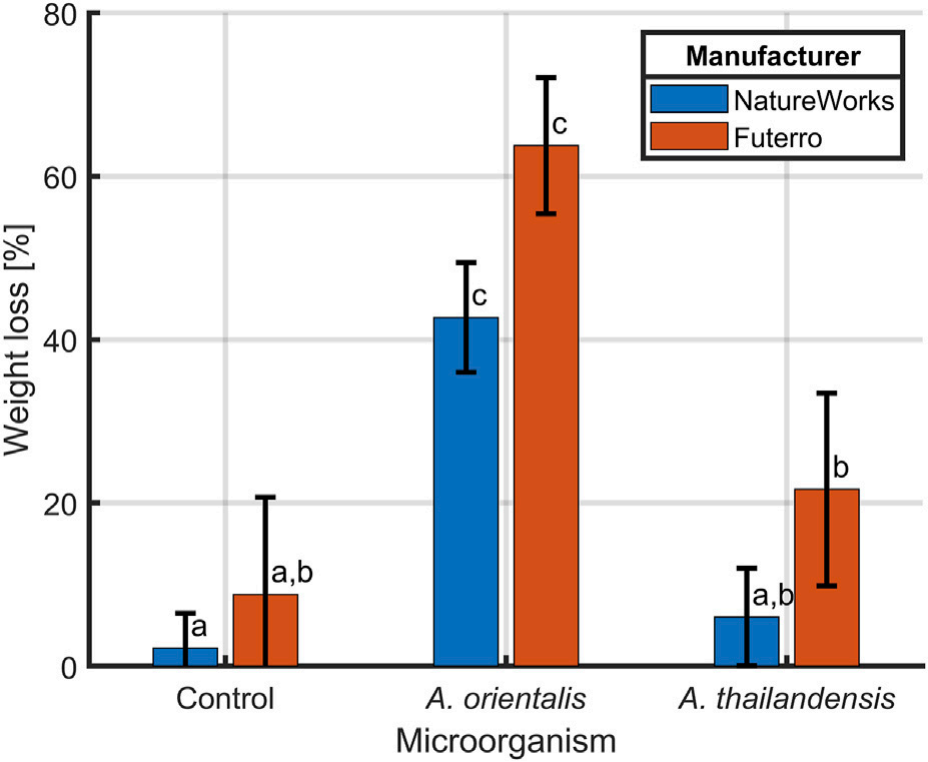
Biodegradation of PLA from two manufacturers NatureWorks and Futerro by *Amycolatopsis orientalis* or *Amycolatopsis thailandensis* compared to non-biodegraded control samples. The PLA weight loss was evaluated after sonication and separation from biomass using a 40 μm cell strainer. Bars bearing different letter indices have a statistically different mean (*p* < 0.05).

The combined application of 120 s of sonication and cell strainers for separating degraded PLA samples was validated in a biodegradation case study. In this study, two PLA types from different manufacturers (NatureWorks and Futerro), were incubated in the presence of *A. orientalis* and *A. thailandensis* for biodegradation. Figure 7 depicts the comparison of gravimetric weight loss after an incubation period of 4 weeks for both the biodegraded samples and control samples. Although for *A. thailandensis* there appears to be some increase in the weight loss compared to the control samples, there is no statistical difference between the untreated samples from either manufacturer and the respective *A. thailandensis*-biodegraded samples. When using *A. orientalis* on the other hand, there is a statistically significant increase in weight loss to 43% and 64% for NatureWorks and Futerro PLA, respectively. However, there was no statistical difference between any of the results from the two manufacturers under the same treatment conditions. The small quantity of weight loss in control samples (2% and 9%) is in line with results from the optimization study of the sonication time. As such, this is likely due to some losses of particles during sample processing.

Whereas plastic biodegradation has been broadly studied, until now there have been limited efforts in studying microbial degradation of powdered samples. Instead, most research efforts focus on the use of granulated particles or extruded films that represent waste fractions and are easier to retrieve from samples (Jarerat and Tokiwa, 2001; Jarerat and Tokiwa, 2003). The milling of plastics to powders is however considered as pretreatment step that can significantly enhance biodegradation by increasing the accessible surface area (Yasin et al., 2022). In cases where powdered PLA has been used, the biodegradation process was monitored through different degradation parameters, such as carbon dioxide production and biological oxygen demand, which indicate the amount of carbon that was transformed into carbon dioxide and the amount of mineralized oxygen, respectively (Benn and Zitomer, 2018; Choe et al., 2022; García-Depraect et al., 2022; López-Ibáñez and Beiras, 2022). While these measurements provide information on the final stage of the biodegradation process, i.e., biomineralization, the weight loss method is suitable for monitoring the first steps of biodegradation, i.e., until biofragmentation (Yasin et al., 2022). Therefore, the developed method plays a crucial role in enabling researchers to obtain a better view of the individual stages of the biodegradation process when working with powdered plastics.

### 3.3 Monitoring the biodegradation process

Measuring biogas production serves for monitoring the final stage of the biodegradation process to the end products, i.e., biomineralization. In this study, biogas production was monitored according to the standard method ISO 14 852 for aerobic plastic biodegradation by determining the carbon dioxide production. In combination with the separation method that was proposed, a carbon balance was calculated by considering the carbon conversion throughout the biodegradation process.

#### 3.3.1 CO_2_ evolution

**FIGURE 8:**
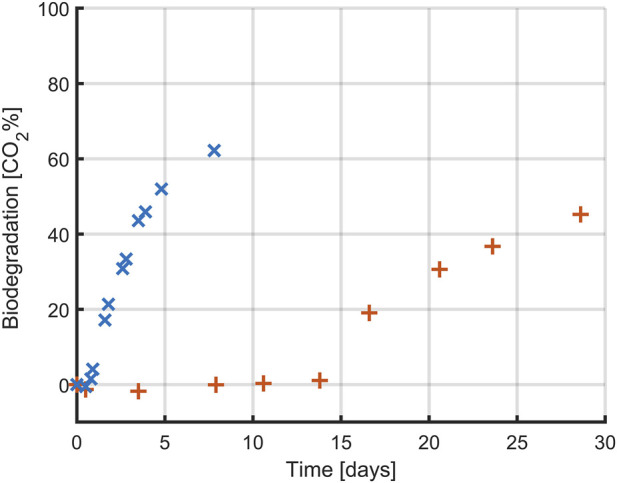
Biodegradation profile (expressed as percentual conversion into CO_2_) of glucose (**x**) and PLA (**+**) by *Amycolatopsis orientalis*. Each data point corresponds to the average of triplicate assays (except for blank, which was prepared in duplicate).

The production of biogas by *A. orientalis* on was studied when using either glucose or PLA as a sole carbon source for respectively 7 and 30 days. Glucose was used as an easily accessible carbon source to validate the implementation of the CO_2_ monitoring method. PLA on the other hand was used to confirm the application of this method for monitoring the various biodegradation phases of bioplastics and to construct a carbon balance. *A. orientalis* was selected for the experiments in this section following the promising results when studying the weight loss of PLA during biodegradation. The profiles for the cumulative production of CO_2_ by *A. orientalis* based on the two carbon sources are presented in Figure 8. These profiles are the result of comparing the averages of 3 samples for each carbon source with 2 blanks, where no carbon source was present.

In both conditions, the highest conversion rate of carbon to CO_2_ was approximately 60%, but this conversion happened faster when glucose was used as carbon source. In the case of glucose, CO_2_ production was quantifiable within the first day of experimentation whereas for the PLA samples it took over 15 days before the first CO_2_ production could be measured. For both carbon sources, CO_2_ production occurred in a close to linear process after this lag period, although at a slower rate for the PLA samples. In the case of glucose, the CO_2_ production rate decreased towards the end of the experiment, indicating that all carbon had been consumed. However, in the case of PLA the final samples still followed a linear trend, indicating that there is more PLA present that could be biodegraded. As such, a full biodegradation process would have taken even longer than 30 days. However, since the goal of the PLA-based experiments was to construct a carbon balance, full conversion of all PLA was not desirable.

Whereas glucose is readily accessible for uptake by the *A. orientalis* metabolism, as seen from the from the short delay until CO_2_ production, this is not the case for PLA. Several steps are required before biopolymers can be used by microorganisms, as the polymer size and semi-crystalline structure hinder microbial attacks (Yasin et al., 2022). As the PLA in these experiments had a powder form with a large specific surface area, it can be assumed that untreated PLA material with a much smaller specific surface area would lead to an even longer time before CO_2_ production and that it would occur at a slower rate. The slow biodegradation rate of PLA in aqueous media has been commonly reported. In separate studies, only 2.0%–8.7% of PLA was degraded after incubation of 28–365 h in the presence of microbial community extracted from wastewater plants (Massardier-Nageotte et al., 2006; Choe et al., 2022) and in artificial aquatic system (Bagheri et al., 2017). The type of microbial inoculum plays a key role in the degradation of polymers. In this study, *A. orientalis* was used as it is a well-studied PLA-degrading bacterium that secretes serine-like protease to degrade PLA. Given that this process is marked by a slow CO_2_ production rate, it is particularly suitable for monitoring with a cumulative measurement method as described by ISO 14 852.

#### 3.3.2 Carbon balance

**FIGURE 9:**
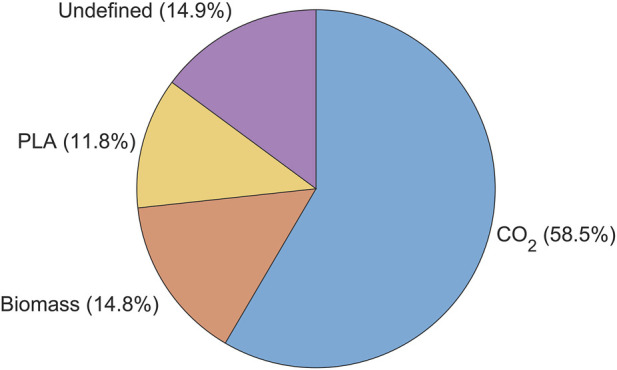
Carbon mass balance % represented by the amount of initial carbon content converted into, biomass (C-Biomass), carbon dioxide (C-evolved CO_2_), residual (C- PLA residual) and undefined carbon when using *Amycolatopsis orientalis* for PLA biodegradation.

To assess the progress of the various phases of PLA biodegradation, a carbon balance was computed based on the experiments conducted with *A. orientalis* in the previous section. Figure 9 illustrates percentage of carbon from the initial quantity of PLA that 1) has been converted to CO_2_, 2) has been converted to biomass, 3) remains as undegraded PLA and 4) is unaccounted for. From this analysis, 85.1% of the carbon from the initial PLA was successfully tracked and measured. More than half (58.5%) of the initial carbon content from PLA was mineralized into biogas, which in this case was CO_2_. Meanwhile, 14.8% of the PLA led to the growth of additional biomass. Finally, 11.8% of the PLA was retrieved from the bioreactor on the final day of sampling. This sample represents the undegraded mass of PLA, which was separated and measured using the method described in Section 2.4.

By using a minimal medium containing only inorganic salts, the only carbon source in the samples was PLA. As expected, a higher biofragmentation rate of PLA was observed compared to the amount converted into CO_2_. On the one hand, the former process reflects the stage of biodegradation in which PLA powder is fragmented into smaller particles by microbial attack. Any particle size larger than the cell strainer was retained, whereas particles smaller than 40 µm passed through and were not accounted for in the residual PLA fraction. Considering that PLA powder of 125–250 μm was used, any particle that passed the strainer had already undergone substantial size reduction. Thus, particle sizes smaller than 40 μm, oligomers and monomers appear in the fraction of unaccounted carbon.

On the other hand, the cumulative CO_2_ production only represents carbon that has been fully biodegraded until the final stage of mineralization (García-Depraect et al., 2022). Any PLA fractions that are larger than 1,000 Da cannot be taken up into the cell and are therefore not yet accessible for mineralization (Sander et al., 2023). As such, the fraction of PLA smaller than 40 µm and larger than 1,000 Da are considered to be the product from the biofragmentation that are broken down further in the bioassimilation step. This PLA fraction forms the unaccounted fraction of PLA, together with dissolved inorganic and organic carbon. Given that the degradation of larger PLA particles to oligomers, dimers, and monomers is slower than the enzymatic degradation of monomers to CO_2_, it can be considered that the PLA fraction undergoing assimilation makes up the majority of the carbon that was unaccounted for.

## 4 Conclusion

This work proposes novel approaches for monitoring the viable cell density and polymer gravimetric weight loss during PLA biodegradation by *A. orientalis* and *A. thailandensis.* First, it was demonstrated that the total population of viable cells can be quantified more accurately by performing a 10 s sonication treatment before diluting and plating the sample. Secondly, powdered PLA was separated from biomass by performing a 120 s sonication treatment that breaks all cell agglomerates and removes attached cells from the PLA surface followed by a filtration step that retains the PLA on a 40 μm cell strainer while letting biomass pass through. Finally, the ISO 14 852 method was implemented to measure the cumulative production of CO_2_ as a function of time. When combining these methods, an overview is obtained of the carbon balance consisting mostly of 1) CO_2_, ii) biomass, 3) PLA that is undergoing the biofragmentation stage to form particles of a size less than 40 µm and 4) PLA that is undergoing the bioassimilation stage towards fractions of about 1,000 Da that can be taken up within the cell membrane.

As such, integrating these newly proposed and existing methods leads to an improved quantifying and understanding of the various stages and aspects of PLA biodegradation by *Amycolatopsis* species.

## Data Availability

The original contributions presented in the study are included in the article/supplementary material, further inquiries can be directed to the corresponding author.
